# Direct Activation of ATM by Resveratrol under Oxidizing Conditions

**DOI:** 10.1371/journal.pone.0097969

**Published:** 2014-06-16

**Authors:** Ji-Hoon Lee, Zhi Guo, Logan R. Myler, Suting Zheng, Tanya T. Paull

**Affiliations:** 1 The Howard Hughes Medical Institute, The Department of Molecular Biosciences, and the Institute for Cellular and Molecular Biology, The University of Texas at Austin, Austin, Texas, United States of America; 2 Department of Medicine, Division of Genetics, Brigham and Women's Hospital, Boston, Massachusetts, United States of America, and the Department of Genetics, Harvard University Medical School, Boston, Massachusetts, United States of America and the Howard Hughes Medical Institute, Brigham and Women's Hospital, Boston, Massachusetts, United States of America; University of Oxford, United Kingdom

## Abstract

Resveratrol has been widely reported to reduce cancer progression in model systems and to selectively induce cell death in transformed cell lines. Many enzymes have been reported to respond to resveratrol in mammalian cells, including the Ataxia-Telangiectasia Mutated (ATM) protein kinase that acts in DNA damage recognition, signaling, and repair. Here we investigate the responses of ATM to resveratrol exposure in normal and transformed human cell lines and find that ATM autophosphorylation and substrate phosphorylation is stimulated by resveratrol in a manner that is promoted by reactive oxygen species (ROS). We observe direct stimulatory effects of resveratrol on purified ATM in vitro and find that the catalytic efficiency of the kinase on a model substrate is increased by resveratrol. In the purified system we also observe a requirement for oxidation, as the effect of resveratrol on ATM signaling is substantially reduced by agents that prevent disulfide bond formation in ATM. These results demonstrate that resveratrol effects on ATM are direct, and suggest a mechanism by which the oxidizing environment of transformed cells promotes ATM activity and blocks cell proliferation.

## Introduction

Resveratrol (trans-3,4′,5-trihyrodoxystilbene) is a naturally-occurring phenolic compound that is well-known for its cardioprotective, anti-carcinogenic, anti-inflammatory, and anti-aging properties in animal model systems [1], [2]. In cancer cells, resveratrol inhibits cell cycle progression, induces apoptosis, and affects autophagy through multiple mechanisms [3], . Resveratrol also has been reported to inhibit tumor invasion and angiogenesis by controlling matrix metalloproteinases, vascular endothelial growth factor, and a number of kinases involved in cell growth control [5]. The mechanism of resveratrol action has been widely debated and attributed to many targets, including SIRT1, cyclooxygenase 1, and AMP-activated protein kinase [6], [7]. Although resveratrol is generally considered to be a anti-oxidant because it induces anti-oxidant enzymes including superoxide dismutase and glutathione S-transferase [8], other reports have indicated pro-oxidant effects that initiate growth arrest and senescence in cancer cells [9], [10].

Ataxia Telangiectasia Mutated (ATM) is a serine/threonine kinase that is activated by DNA damage through interactions with the Mre11/Rad50/Nbs1 (MRN) complex that recognizes double-strand breaks in DNA and activates the kinase at damage sites [11], [12]. ATM can also be activated in the absence of MRN or DNA damage by direct oxidation and generation of disulfide bonds within the homodimer complex [13], [14]. ATM phosphorylates numerous downstream target proteins that are involved in cell cycle checkpoint activation, DNA repair, and apoptosis [15], [16] and affects many diverse cellular processes including autophagy, senescence, and mitochondrial functions [17].

A link between resveratrol and ATM has emerged in recent years from studies suggesting that some of the effects of resveratrol on cell cycle arrest and apoptosis take place through an ATM-dependent signaling pathway [9], [18]–[21]. Incubation of human cancer cell lines with resveratrol was shown to lead to S phase cell cycle arrest or senescence that could be blocked by caffeine (an inhibitor of both ATM and the related ATR protein kinase) or by an ATM-specific inhibitor [9], [18]. In addition, resveratrol was shown to stimulate ATM autophosphorylation as well as phosphorylation of p53 and Nbs1, but not in caffeine-treated cells or in cells lacking ATM [19]. In this study, resveratrol did not efficiently induce p53 phosphorylation in Nijmegen breakage syndrome (NBS) cells that lack wild-type MRN complex even though ATM autophosphorylation was stimulated, suggesting that resveratrol is upstream of the MRN complex and the MRN complex is required for efficient signal transduction to ATM downstream. In some of these studies γ-H2AX was observed in response to resveratrol treatment, suggesting that resveratrol either induces DNA damage or potentiates it with higher levels of reactive oxygen species (ROS) [18], [21], although the mechanism of ATM stimulation by resveratrol, as with many proposed resveratrol targets, is unknown. In this work, we focus on the effects of resveratrol on ATM-dependent phosphorylation events in human cell lines as well in a reconstituted enzyme assay in vitro. Surprisingly, we find that resveratrol appears to directly activate ATM in both contexts and requires an oxidizing environment to exert these effects.

## Materials and Methods

### Reagents

Commercial reagents included resveratrol (Sigma, R5010-100 mg), KU-55933 (EMD, 80017-420), TCEP (Pierce, 20490), NAC (Fisher Scientific, 01049-25), genistein (Sigma, G6649-25MG), and piceatannol (Sigma, P0453-5MG).

### Cell culture and damage treatments

Human HEK293T (ATCC), HCT116 (ATCC), and GM08399 (Coriell) cells were cultured in Dulbecco's Modified Eagle Medium (DMEM, Invitrogen) supplemented with 10% fetal bovine serum (FBS)(Invitrogen). Cells were treated with resveratrol (0.1 mM) as indicated in the figure legends, in DMEM media without FBS. Treatments with H_2_O_2_ or bleomycin were for 30 minutes immediately before harvesting. Preparation of ATM shRNA lentivirus: 293T cells were cotransfected for 12 h with ATM shRNA plasmid (sc-29761-SH, Santa Cruz Biotechnology) and the lenti-viral packaging constructs VSVG and Delta 8.9 (ratio = 2.5 ATM shRNA plasmid: 1.5 Delta 8.9: 1 VSVG) using lipofectamine 2000 reagent (Invitrogen) according to manufacturer instructions. 48 and 72 hours after transfection, the medium containing virus was collected, pooled, and filtered using a 0.45 µM syringe filter. The virus was then applied to the GM08399 fibroblasts, cultured for 24 hours, followed by selection with puromycin (1 µg/ml) (Invitrogen) for 3 days before testing for ATM depletion.

### Immunocytochemistry and microscopy

293T and GM08399 cells were seeded on chamber slides and grown for 48 hr. Cells were incubated in the presence or absence of resveratrol (0.1 mM) for 30 min in DMEM media (Invitrogen) without FBS. Then cells were treated with H_2_O_2_ (0.1 mM) or bleomycin (1 µg/ml) for 30 min and permeabilized with cold 100% methanol for 5 min. Each step was followed by washing with PBS 3 times for 5 min. Cells were incubated with 8% bovine serum albumin (BSA) in PBS for 1 hr and washed with PBS for 5 min. The primary antibody against γ-H2AX (GTX80694, Genetex) was diluted 1∶750 in 1% BSA/PBS and added for 1 hr, followed by washing with PBS 3 times for 5 min. The secondary antibody against mouse IgG Alexa Fluor 488 (A21202, Invitrogen) was diluted 1∶500 in 1% BSA/PBS and added for 30 min, followed by washing with PBS 3 times for 5 min. Cells were counterstained with 4′,6-diamino-2phenylindole (DAPI) (D3571, Invitrogen) at 1∶30,000 for 5 min in PBS. Cells were washed with PBS 3 times for 5 min and washed with water. Chambers were separated and cover slips were placed after adding one drop of gold antifade (P36934, Invitrogen). For foci counting, cells were imaged on a Zeiss Axiovert 200M fluorescence microscope with 100W Hg lamp excitation using AxioVision software. For images of cells, a Zeiss LSM 710 confocal microscope was used, controlled by Zen software. The number of foci and γ-H2AX signal in nucleus were analyzed with ImageJ.

### Comet assay

GM08399 cells were treated with resveratrol (0.1 mM) or bleomycin (1 µg/ml) as indicated in the figure legend for 30 min before harvesting. Alkaline comet assays were performed using OxiSelect comet assay kit (STA-350, Cell Biolabs) following the manufacturer's protocol. Samples were observed under a Zeiss Axiovert 200M fluorescence microscope.

### Western Blotting

Cells were lysed in cell lysis buffer (9803, Cell Signaling) and lysate (10 µg) was separated by SDS-PAGE and analyzed by western blotting. Proteins were transferred to PVDF-FL membrane (Millipore) and probed with antibodies directed against ATM (GRX70103, Genetex), phospho-ATM Ser-1981 (AF-1655, R&D Systems), p53 (GTX70214, Genetex), phospho-p53 Ser-15 (9286, Cell Signaling), Smc1 (4802, Cell Signaling), phospho-Smc1 Ser-957(4805S, Cell Signaling), Kap1 (ab22553, Abcam), phospho-Kap1 Ser-824 (A300-767A, Bethyl Laboratories), Nbs1 (GTX70224, Genetex), phospho-Nbs1 Ser-343 (ab47272, Abcam), Chk2 (GTX70295, Genetex), and phospho-Chk2 Thr-68 (2661S, Cell Signaling) followed by detection with IRdye 800 anti-mouse (Rockland, RL-610-132-121) or Alexa Fluor 680 anti-rabbit (Invitrogen, A21076) secondary antibodies. Western blots were analyzed and quantitated using a Licor Odyssey system.

### ATM kinase activity kinetics

ATM (0.36 nM) was incubated with various concentrations of substrate GST-p53 [22](40, 60, 80, 100, 120, 140, 160, and 320 nM) and H_2_O_2_ (817 µM) in the presence or absence of resveratrol (278 µM) for various incubation times (0, 5, 10, 20, 40, 80, 140, 200, and 240 min). Proteins were transferred to PVDF-FL membrane and probed with antibody directed against phospho-p53 Ser-15 followed by detection with Alexa Fluor 680 anti-rabbit secondary antibody. The signal of phosphorylated p53 in each reaction was quantitated using a Licor Odyssey System and converted to actual concentration of phosphorylated p53 by comparison to a phosphorylated standard, which was determined by quantitation of the phosphorylated product. V_max_ and K_m_ were analyzed using Prism software.

## Results and Discussion

### Resveratrol activates ATM in human cells

Previous experiments have indicated that resveratrol treatment increases levels of ATM activation in human ovarian cancer cell lines [18], HCT116 colon carcinoma cells [9], and in immortalized lymphoblastoid cell lines [19]. These studies showed increased levels of ATM autophosphorylation on Ser-1981 and phosphorylation of Nbs1, p53, and Chk2, suggesting that resveratrol activates ATM to phosphorylate its targets. While these results are largely in agreement, it was unclear how resveratrol activates ATM. One study suggested that resveratrol induces oxidative stress, which is responsible for the activation [9], while other studies have indicated that DNA damage is caused by resveratrol treatment, as measured by γ-H2AX formation [18]. To investigate this phenomenon further, we examined the effects of resveratrol treatment on ATM-related phosphorylation events in the human cell lines HEK293T and HCT116.

**Figure 1:**
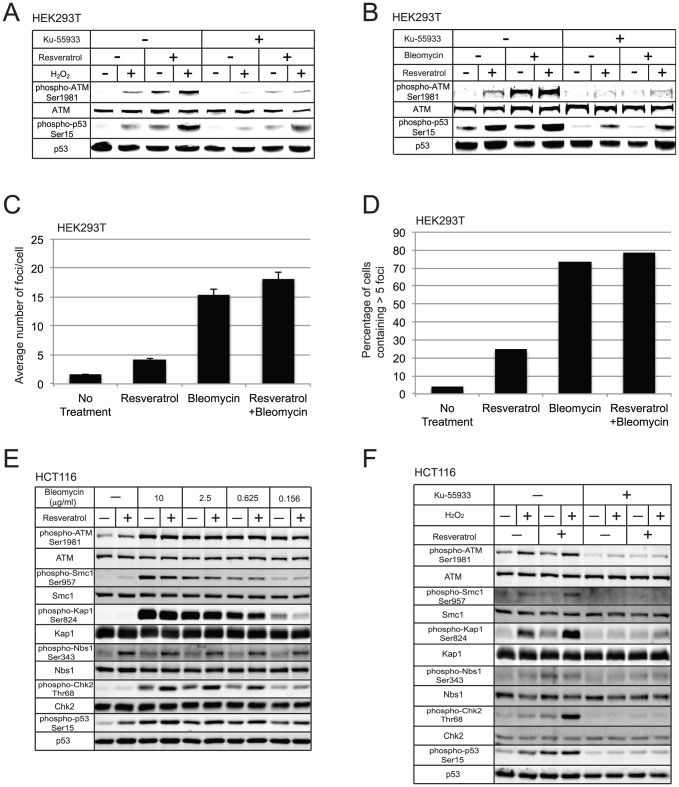
Resveratrol activates ATM in transformed human cell lines. (a, b) Human HEK293T cells were treated with 0.01 mM ATM inhibitor (KU-55933) or vehicle (DMSO) for 1 hour, followed by incubation in media also containing 0.1 mM resveratrol (or DMSO), as indicated. After 30 minutes incubation with resveratrol, H_2_O_2_ (0.1 mM) or bleomycin (1 µg/ml) was added as indicated for 30 minutes before harvesting. Western blotting was performed with with antibodies directed against ATM, phospho-ATM Ser-1981, p53, and phospho-p53 Ser-15 as indicated. (c, d) Human HEK293T cells were treated with resveratrol, bleomycin, or both as in (a) and probed for γ-H2AX foci by immunofluorescence. Cell images (291, 267, 216, and 260 cells, respectively) were analyzed for foci using Image J software, and the average number of foci per cell as well as the percentage of cells containing >5 foci were quantitated. (e, f) Human HCT116 cells were incubated with KU-55933, resveratrol, H_2_O_2_, and bleomycin as in (a, b) but additional phosphorylation targets were examined using antibodies directed against Smc1, phospho-Smc1 Ser-957, Kap1, phospho-Kap1 Ser-824, Nbs1, phospho-Nbs1 Ser-343, Chk2, and phospho-Chk2 Thr-68 as indicated.

As shown in Fig. 1A and B, resveratrol treatment does induce ATM autophosphorylation on ser1981 as well as phosphorylation of p53 on ser15 in HEK293T cells, consistent with previous reports. Treatment of cells with bleomycin to induce DNA double-strand breaks or H_2_O_2_ to increase oxidative stress also induced this effect, and treatment with both resveratrol and bleomycin or resveratrol and peroxide increased ATM autophosphorylation and p53 phosphorylation by 2.5-fold +/−0.6 relative to either treatment alone. These phosphorylation events were largely blocked by treatment of cells with an ATM-specific inhibitor (KU-55933) [23], indicating that they are ATM dependent (Figure 1A, B). Taken together, these results demonstrate that resveratrol stimulates ATM kinase activity by itself and also augments the activation of ATM during DNA damage or oxidative stress in these cells.

A previous study showed that histone H2AX is phosphorylated upon resveratrol exposure [18], which is generally interpreted as a sign of DNA double-strand break formation [24]. To investigate whether resveratrol also induces breaks under our experimental conditions, we analyzed γ-H2AX formation in HEK293T cells and found that there is a measurable increase in the number of foci per cell and in the number of cells in a population exhibiting 5 or more γ-H2AX foci per cell in response to resveratrol exposure (Fig. 1C, D). Bleomycin treatment was used as a positive control in the experiment, which induced a much higher level of γ-H2AX foci per cell.

To extend these results, we used the colon carcinoma cell line HCT116 and analyzed phosphorylation of Smc1, Kap1, Nbs1, and Chk2 in addition to ATM and p53 phosphorylation (Fig. 1E). In these cells, resveratrol treatment alone also stimulated phosphorylation of p53 and Nbs1, as well as ATM autophosphorylation. Titration of bleomycin induced the phosphorylation of all the ATM targets as well as autophosphorylation, but there was little additional effect of resveratrol apart from a ∼2-fold increase in Chk2 thr68 phosphorylation, and other phosphorylation events (Kap1, SMC1) were unaffected by resveratrol treatment. In contrast, simultaneous treatment with H_2_O_2_ yielded a different outcome: autophosphorylation of ATM was unaffected by resveratrol but phospho-Kap1, phospho-Smc1, and phospho-Chk2 were increased by 3-fold (Fig. 1F). Incubation with the ATM inhibitor KU-55933 inhibited all of these phosphorylation events. Thus resveratrol stimulates ATM-dependent phosphorylation of several different targets in HCT116 cells. Some targets are phosphorylated in the presence of resveratrol alone, while others are phosphorylated only with simultaneous oxidative stress. This difference was not due to the magnitude of damage elicited by the two different forms of stress, since resveratrol also did not show cooperative effects with low levels of bleomycin in this cell line (Fig. 1E).

**Figure 2:**
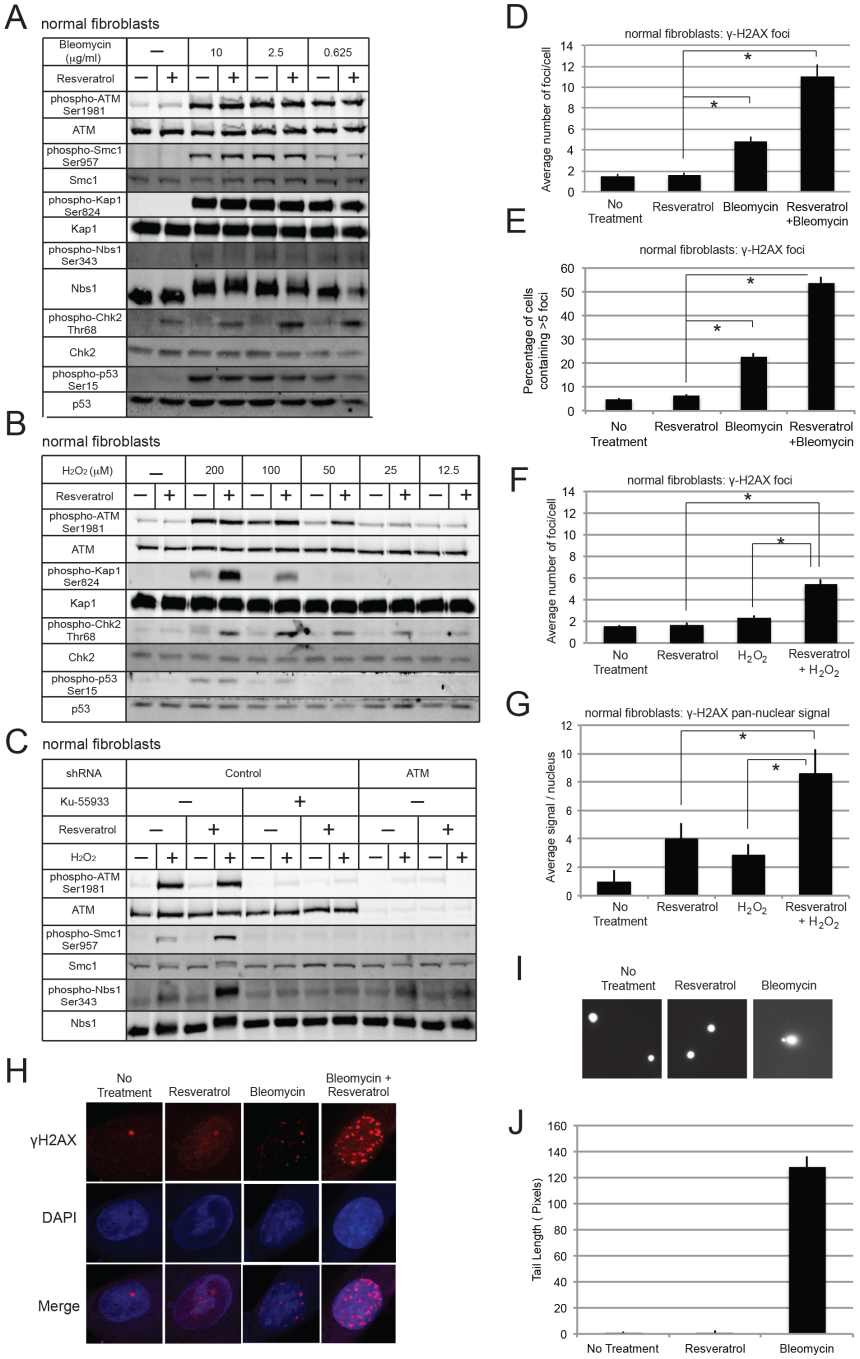
Resveratrol activates ATM in human primary fibroblasts (GM08399) in combination with H_2_O_2_ or bleomycin. (a, b) The effects of resveratrol on human primary fibroblasts were tested as in Figure 1 but with varying levels of H_2_O_2_ or bleomycin as shown. (c) To deplete ATM, the fibroblasts were transduced with lentivirus expressing shRNA directed against ATM shRNA plasmid. After selection with puromycin, cells were tested for ATM expression and ATM target phosphorylation in combination with KU-55933, resveratrol, H_2_O_2_, and bleomycin as in Fig. 1. (d) Human primary fibroblasts were treated with resveratrol, bleomycin, or both as in (a) and probed for γ-H2AX foci by immunofluorescence. Cell images (82, 83, 78, and 82 cells, respectively, were analyzed for foci using Image J software, and the average number of foci per cell were quantitated. Error bars show standard error and * indicates comparisons in which p<0.05. (e) Human primary fibroblasts were treated as in (d) and the percentage of cells containing >5 foci was quantitated. Cell images from 3 independent experiments with a total of 266, 264, 248, and 269 cells, respectively, were quantitated. (f) Human primary fibroblasts were treated with resveratrol (100 µM), hydrogen peroxide, or both as in (b) and were quantitated for γ-H2AX foci by immunofluorescence. 107, 110, 104, and 106 cells, respectively, were analyzed for foci using Image J software, and the average number of foci per cell was calculated. (g) Quantitation of total pan-nuclear γ-H2AX signal per nucleus in cells treated with resveratrol, H_2_O_2_, and bleomycin as in Fig. 1. The average nuclear signal in untreated cells was normalized to 1. (h) Representative immunofluorescence images with fibroblasts treated as in (a). (i) Representative comet assay images with fibroblasts treated as in (a). (j) Quantification of comet tail length from fibroblasts treated as in (a); 30 cells were measured for each condition.

To determine if these observations using transformed cells also apply to normal cells, we used untransformed human fibroblasts (GM08399)(Fig. 2). The levels of phosphorylation on ATM targets were largely unchanged in response to resveratrol treatment in these cells, with the exception of a 2.5-fold increase in phosphorylated Chk2 (Fig. 2A). A titration of resveratrol in these cells shows a dose-dependent increase (Fig. S1). Similar to the observations in HCT116 cells, DNA damage induced by bleomycin treatment strongly induced phosphorylation of ATM itself as well as Smc1, Kap1, Nbs1, and p53, yet resveratrol had no discernible effect on these modifications apart from the effect on Chk2 (Fig. 2A). In contrast, resveratrol strongly stimulated Kap1 and Smc1 phosphorylation by 6-fold when given simultaneously with hydrogen peroxide (Fig. 2B, C), and the magnitude of the increase in the phosphorylation events was dependent on both the level of peroxide treatment as well as resveratrol. All of these phosphorylation events are dependent on ATM, since treatment with KU-55933 or depletion of ATM protein by shRNA eliminated the phosphorylation (Fig. 2C). We do not know why there is a much stronger effect of resveratrol on some substrates compared to others; it is possible that this is related to the affinity of some substrates for ATM, similar to what we have observed for effects of MRN [22], [25].

We also examined γ-H2AX foci in the normal fibroblasts and found that, in contrast to the transformed cells, resveratrol treatment alone did not induce an increase in γ-H2AX foci, examining both the average number of foci per cell as well as the percentage of cells containing 5 or more foci (Fig. 2D). However, resveratrol treatment increased the number of γ-H2AX foci observed by 2 to 3-fold when given simultaneously with either bleomycin or peroxide treatment (Fig. 2D, E, F). A titration of resveratrol also shows a dose response in the number of γ-H2AX foci observed per cell (Fig. S2).

It should be noted here that the quantitation of the immunofluorescence images was performed using Image J-derived software to count individual foci based on a set of training images. Using this software, we also analyzed total pan-nuclear γ-H2AX signal per cell, not counting discrete spots but general staining intensity, in comparison to the background level of γ-H2AX in untreated cells. These results show that resveratrol treatment alone does increase γ-H2AX signal in a pan-nuclear pattern but not in discrete foci (Fig. 2G; examples of images shown in Fig. 2H). This is interesting as it suggests a global activation of ATM, not localized to damage sites, and is reminiscent of pan-nuclear ATM autophosphorylation observed with treatments that are thought to alter chromatin structure [26]. We do not think that this increased γ-H2AX is associated with DNA damage, as comet assays showed no sign of chromosomal DNA fragments with resveratrol treatment (Fig. 2I, J).

Overall, these results show that the responses in all the cell lines were similar in that resveratrol had moderate effects on ATM phosphorylation events when given with DNA damage, but showed much higher stimulation when exposed simultaneously with peroxide. In comparison, the HEK293T cells exhibited more responsiveness to DNA damage in the absence of oxidative stress. However, since some transformed cell lines are known to have higher levels of ROS compared to normal cells, it is possible that higher ROS in HEK293T cells promotes the resveratrol response to DNA DSBs (see below).

### Direct activation of ATM by resveratrol in vitro

**Figure 3:**
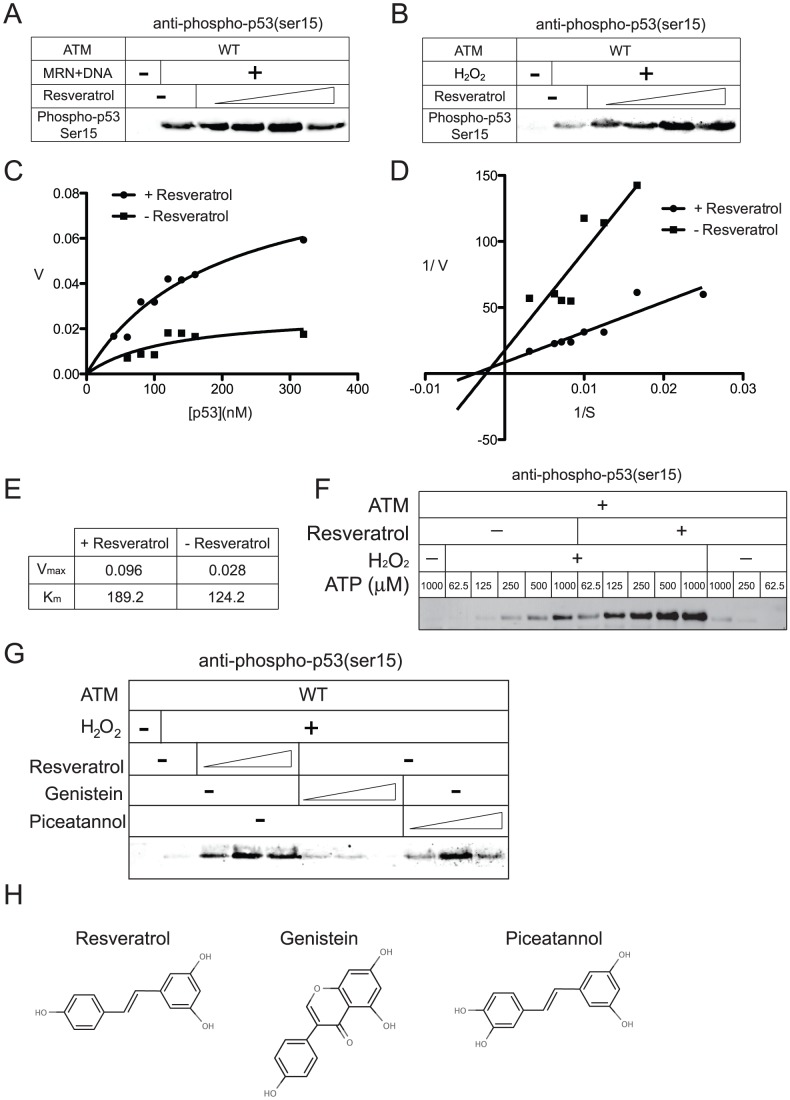
Purified ATM is stimulated by resveratrol in vitro. (a) MRN/DNA-dependent ATM activity was tested with 0.36 nM ATM, 2.2 nM MRN, 50 nM GST-p53, and 10 ng (∼140 nM) linear DNA in a 40 µl reaction as described previously [25]. (b) H_2_O_2_-dependent ATM activity was performed with 817 µM H_2_O_2_ in vitro as described previously [13] in the presence of 0, 69.5, 139, 278, or 556 µM resveratrol. (c) ATM kinase assays were performed with 0.36 nM ATM, 817 µM H_2_O_2_, and varying concentrations of GST-p53 substrate (40, 60, 80, 100, 120, 140, 160, and 320 nM) as indicated, in the presence or absence of 278 µM resveratrol. Phosphorylated p53 was quantitated using western blotting in comparison to standards, and the rate of phosphorylation (nmoles/min/pmole ATM) is plotted as a function of p53 substrate concentration (d) Skatchard plot is shown based on data in (c). (e) V_max_ (nmoles/min/pmole ATM) and K_m_ (nM) values calculated from data shown in (d) and (e). (f) ATM kinase assay as in (a) with 817 µM H_2_O_2_, 278 µM resveratrol, and varying levels of ATP as indicated. (g) ATM kinase assays were performed as in (a) except with 100, 278, and 830 µM resveratrol, genistein, or piceatannol in the presence of H_2_O_2_. (h) diagrams of resveratrol, genistein, and piceatannol structures.

To determine if the effects of resveratrol on ATM are direct and whether they require oxidation, we used an in vitro kinase assay with purified components. As we have shown previously, recombinant dimeric ATM can be activated over 100-fold by the addition of the MRN complex and linear DNA [25] or by the addition of oxidizing reagents such as H_2_O_2_ [13]. Here we tested the effects of resveratrol on ATM using GST-p53 as a model substrate in vitro, assessing kinase activity with phospho-specific antibody directed against ser15 and analyzing the reactions with quantitative western blotting. We found that resveratrol does stimulate ATM kinase activity by itself and also increases the level of p53 phosphorylation in the presence of either the MRN complex and DNA or in the presence of H_2_O_2_ by 2 to 3-fold (Fig. 3A, B), similar to the observations in HCT116 and normal human fibroblasts. Since ATM is activated by resveratrol in the reactions with H_2_O_2_, in the absence of MRN or DNA, it is clear that DNA damage is not essential for ATM stimulation by resveratrol.

To determine the mechanism of resveratrol stimulation of ATM, an analysis of ATM phosphorylation kinetics was performed using peroxide as the primary stimulant, measuring the effects of resveratrol on the rate of phosphorylation using quantitative western blotting of phospho-p53 (Fig. 3C, D). These results (summarized in Fig. 3E) show that resveratrol does not improve the affinity of ATM for its substrate since the K_m_ was 124.2 nM in the absence of resveratrol and 189.2 nM in the presence of resveratrol. However, the maximum reaction rate (V_max_) was 3.5-fold higher in the presence of resveratrol: 6.4 nmoles/min/pmole of ATM compared to 1.9 nmoles/min/pmole of ATM in the absence of resveratrol, indicating that resveratrol increases ATM catalytic efficiency.

We also analyzed the effects of ATP concentration on resveratrol effects on ATM, and found that resveratrol activates ATM more efficiently under limiting ATP conditions (Fig. 3F). While the increase in substrate phosphorylation seen with resveratrol is ∼3-fold in the presence of 1 mM ATP (our standard reaction conditions), the fold increase in substrate phosphorylation in comparison to the reactions without resveratrol are 6.1, 7.3, and 9.0-fold at 500, 250, and 125 µM ATP, respectively. The overall level of phosphorylation is higher with higher levels of ATP but the fold stimulation by resveratrol is greater when ATP is limiting.

Resveratrol is one of several natural phenolic compounds that have been shown to have biologically relevant properties in mammalian cells. For instance, genistein is in the class of isoflavonoids and has also been shown to induce ATM kinase activity in human cells [27], [28]. Piceatannol, a hydroxylated analogue of resveratrol, also shows very similar effects to resveratrol in cultured cells and animal models, including anti-oxidant and anti-cancer properties [29]. Here we compared both genistein and piceatannol to resveratrol in the ATM kinase activity in vitro and found that piceatannol had very similar effects on ATM-dependent phosphorylation events in the presence of H_2_O_2_ in vitro but genistein did not affect ATM activity (Figure 3G). Neither compound induced ATM activation in the absence of H_2_O_2_ or DNA damage (Fig. S3). Since genistein has been reported to act as a topoisomerase poison [30], and markers of DNA damage were observed in cells treated with genistein [28], it is likely that this compound activates ATM indirectly by inducing topoisomerase-generated DNA breaks. In contrast, piceatannol appears to function similarly to resveratrol and is identical in structure apart from the additional hydroxyl group (Fig. 3H).

### ATM stimulation by resveratrol requires oxidation of ATM

**Figure 4:**
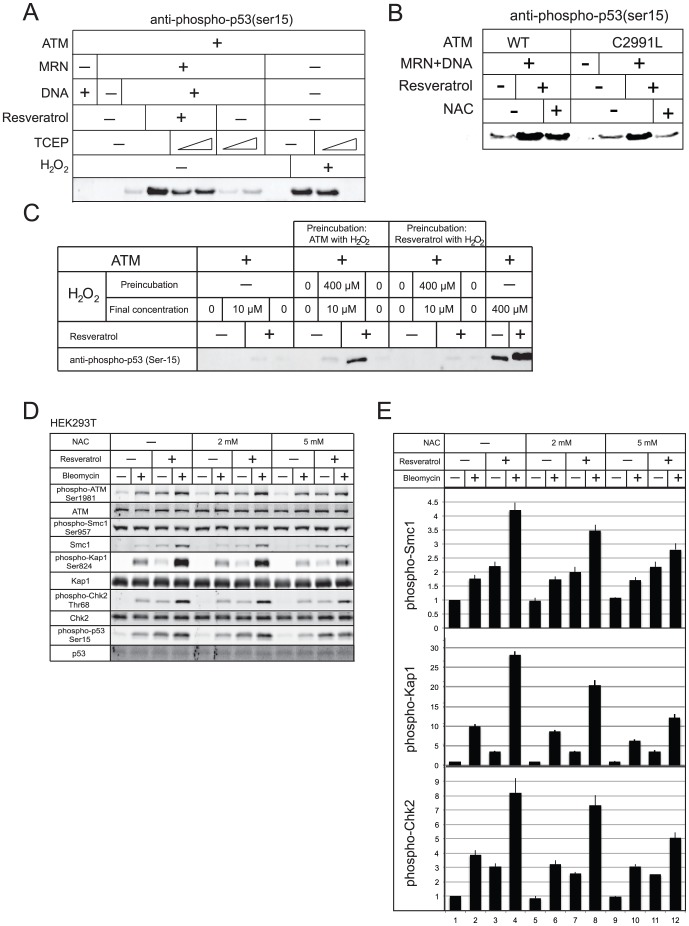
Oxidation is required for resveratrol stimulation of ATM. (a) ATM kinase assays were performed as in Fig. 3 except with 0.5 and 2.5 mM TCEP as indicated. (b) ATM kinase assays were performed as in Fig. 3 except with 0.36 nM ATM mutant (C2991L) and wild-type proteins as indicated. (c) ATM or resveratrol was pre-incubated with H_2_O_2_ (400 µM) as indicated for 15 min. Samples were diluted 40-fold with kinase reaction buffer containing 200 nM GST-p53 and incubated 1.5 hr. Final concentration of ATM and resveratrol is 0.36 nM and 0.1 mM, respectively, in all reactions. (d) HEK293T cells were preincubated with either 2 or 5 mM NAC as indicated for 16 hrs, followed by treatment with resveratrol and bleomycin as indicated. (e) (Quantitation of phosphorylated substrate levels from 3 independent experiments including those shown in (d); error bars indicate standard deviation.).

ATM activity is strongly affected by levels of ROS, and multiple disulfide bonds form between ATM monomers to create a covalently-linked, active dimer, as previously shown [13]. To determine if ROS are important for ATM stimulation by resveratrol in vitro, the disulfide-specific reducing agent TCEP was added to reactions in which ATM is activated by a combination of MRN, DNA, and resveratrol (Fig. 4A). This experiment does show a reduction in the efficiency of resveratrol-dependent ATM stimulation by TCEP, while it has no effect on the MRN/DNA reaction alone, as shown previously [14]. The higher level of TCEP used here is sufficient to completely block ATM activation by H_2_O_2_ (Fig. 4A). A similar experiment with the antioxidant N-acetyl cysteine (NAC) also showed a reduction in the resveratrol-dependent increase in p53 phosphorylation (Fig. 4B), indicating that there is an effect of oxidation on resveratrol stimulation of ATM but that it is not absolutely required as it is when ATM is activated by oxidation in the absence of MRN and DNA [13]. We previously described a mutant of ATM that is specifically deficient in the oxidation pathway of ATM activation, C2991L [13]. This mutant can be activated normally by MRN/DNA but shows no activity when oxidized because the cysteine that is mutated cannot form the disulfide that is required for activation via oxidative stress. Surprisingly, when the C2991L mutant was tested for MRN/DNA-dependent stimulation, this was increased by the presence of resveratrol, and the increase was also eliminated by NAC (Fig. 4B). These results suggest that while oxidation is important for the resveratrol-dependent increase, this increase does not depend on C2991 oxidation.

To understand what the role of the oxidizing agent is in resveratrol stimulation of ATM, we performed a 2-stage reaction in which we first incubated the peroxide with either ATM or resveratrol in a small volume followed by dilution into the complex reaction containing the remaining reaction components and the p53 substrate (Fig. 4C). The preincubation level of H_2_O_2_ was high (400 µM) but the final diluted concentration (10 µM) was lower than the required amount for in vitro activation. (Note that the high concentration of H_2_O_2_ is required because ATM is stored in the presence of reducing agent to prevent spontaneous activation). The results from this experiment show that preincubation of H_2_O_2_ with ATM in the first reaction promotes subsequent activation of ATM by resveratrol in the second reaction. In contrast, resveratrol preincubation with H_2_O_2_ has no effect. This rules out the possibility that the peroxide is modifying resveratrol in some way, and suggests that ATM oxidation is required for the full stimulatory effects of this compound.

To test this idea further in cells, we again examined resveratrol simulation of ATM phosphorylation events in HEK293T cells in combination with DNA damage induced by bleomycin. Pretreatment of cells with NAC reduced the extent to which resveratrol promotes ATM phosphorylation of its targets Smc1, Kap1, and Chk2 (results from 3 experiments quantified in Fig. 4E), but had little effect on the phosphorylation induced by bleomycin alone.

Overall, these results show that resveratrol directly modulates the activity of ATM and has effects on both the MRN/DNA mechanism of activation as well as the oxidation pathway. In addition, we find that levels of ROS dictate the efficiency of resveratrol effects on ATM, such that high levels of ROS promote activation, both in cell lines and in a purified system in vitro. Since we have previously demonstrated the formation of multiple disulfide bonds in ATM under oxidizing conditions, it is possible that the functional effects of resveratrol require conformational changes dependent on one or more of these disulfide bridges. This dependence on ROS may form at least part of the basis for selective effects of resveratrol on cancer cells versus normal cells as transformation is known to increase levels of ROS [31].

## Supporting Information

Figure S1 Human primary fibroblasts were treated with resveratrol, hydrogen peroxide, or both as in Fig. 2B. The western blot was probed for phospho-Kap1(S824), Kap1, phospho-Chk2(T68), and Chk2 as indicated.
(DOCX)

Figure S2 Human primary fibroblasts were treated with resveratrol, hydrogen peroxide (100 µM), or both as in Fig. 2F. The number of γH2AX foci per cell was quantitated (84, 92, 85, 80, 84, 93, 93, and 88 cells were counted, respectively) and the average number of foci per cell is shown with standard error. * indicates comparisons in which p<0.05.
(DOCX)

Figure S3 ATM kinase assays were performed as in Figure 3G with 100 µM H_2_O_2_, resveratrol (100 µM), genistein (100 µM) or piceatannol (100 µM) as indicated.
(DOCX)
