# Supplementary figures and images for: Direct Activation of ATM by Resveratrol under Oxidizing Conditions
Source: PLoS One. 2014 Jun 16;9(6):e97969. doi: 10.1371/journal.pone.0097969 (PMC4059639; doi:10.1371/journal.pone.0097969)

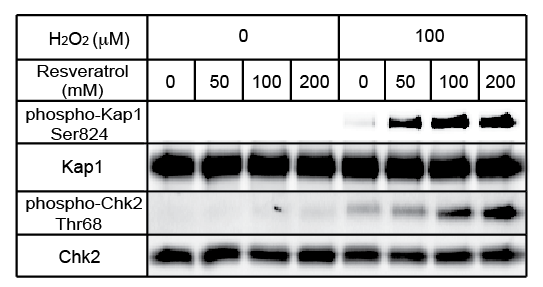

Supplement: Figure S1 — Human primary fibroblasts were treated with resveratrol, hydrogen peroxide, or both as in Fig. 2B. The western blot was probed for phospho-Kap1(S824), Kap1, phospho-Chk2(T68), and Chk2 as indicated. (DOCX) [file pone.0097969.s001.docx]

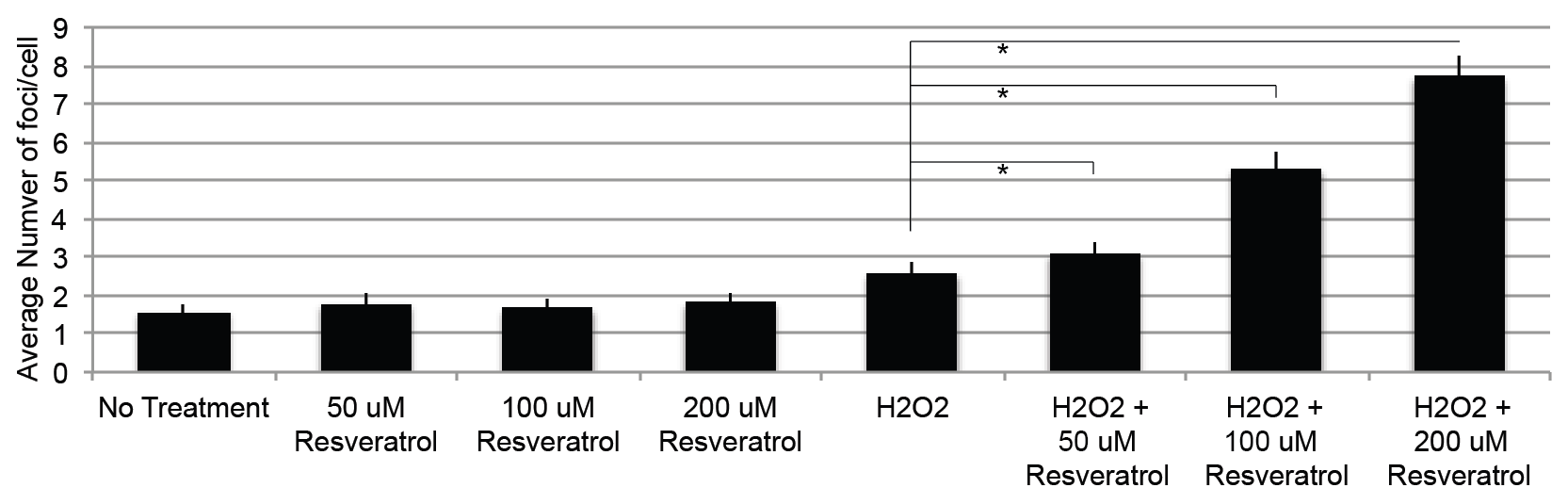

Supplement: Figure S2 — Human primary fibroblasts were treated with resveratrol, hydrogen peroxide (100 µM), or both as in Fig. 2F. The number of γH2AX foci per cell was quantitated (84, 92, 85, 80, 84, 93, 93, and 88 cells were counted, respectively) and the average number of foci per cell is shown with standard error. * indicates comparisons in which p<0.05. (DOCX) [file pone.0097969.s002.docx]

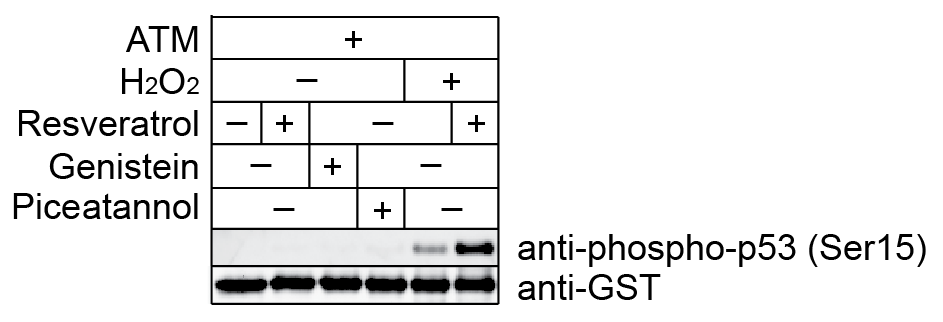

Supplement: Figure S3 — ATM kinase assays were performed as in Figure 3G with 100 µM H2O2, resveratrol (100 µM), genistein (100 µM) or piceatannol (100 µM) as indicated. (DOCX) [file pone.0097969.s003.docx]
